# Corticospinal Tract Hypoperfusion Associated With Unexplained Early Neurological Deterioration After Intravenous Thrombolysis

**DOI:** 10.3389/fneur.2022.854915

**Published:** 2022-03-28

**Authors:** Danfeng Zhang, Wansi Zhong, Luowei Chen, Chao Xu, Shenqiang Yan, Ying Zhou, Xiaodong Ma, Min Lou

**Affiliations:** ^1^Department of Neurology, The Second Affiliated Hospital of Zhejiang University, School of Medicine, Hangzhou, China; ^2^Department of Neurology, Haiyan People's Hospital, Zhejiang, China; ^3^Department of Neurology, Zhejiang Provincial People's Hospital, Hangzhou, China

**Keywords:** acute ischemic stroke, intravenous thrombolysis, corticospinal tract, unexplained early neurological deterioration, computed tomography perfusion

## Abstract

**Background and Purpose:**

Early neurological deterioration (END) occurs in 10% among patients with acute ischemic stroke (AIS) who are receiving intravenous thrombolysis (IVT). Over half of them have no straightforward causes, which is referred to as unexplained END. We aimed to explore whether the presence of baseline corticospinal tract (CST) hypoperfusion could predict the development of unexplained END at 24 h in patients with AIS after receiving IVT.

**Methods:**

We retrospectively analyzed the clinical and imaging data from patients with AIS who received IVT. Unexplained END was defined as ≥ 2-point increase of National Institutes of Health Stroke Scale (NIHSS) from baseline to 24 h without straightforward causes. Hypoperfusion lesions involving CST and other cerebral areas were identified on perfusion maps.

**Results:**

Among 807 patients, CST hypoperfusion and non-CST hypoperfusion occurred in 488 (60.5%) and 319 (39.5%) patients, respectively. Patients with CST hypoperfusion were more likely to have unexplained END compared with patients with non-CST hypoperfusion (16.6 vs. 2.8%, *P* < 0.001). Binary logistics regression analysis showed that CST hypoperfusion was independently associated with unexplained END after IVT (OR = 5.64; 95% CI: 2.699–11.785; *P* < 0.001) after adjusting for baseline NIHSS, onset to needle time, baseline hypoperfusion volume, atrial fibrillation, and hypertension.

**Conclusions:**

Patients with CST hypoperfusion were more likely to suffer from unexplained END after IVT, implying potential mechanisms and potential prevention of unexplained END.

## Introduction

Intravenous thrombolysis (IVT) with recombinant tissue plasminogen activator (rt-PA) has been a standard treatment of acute ischemic stroke (AIS) (1). However, approximately 10% of patients with AIS who received IVT may experience early neurological deterioration (END), an ominous clinical event closely associated with poor outcomes (2). The specific causes of END include symptomatic intracerebral hemorrhage, malignant edema, early recurrent stroke, and post-stroke seizure. However, it is recently reported that 47–70% of END have no clear cause, which is referred to as “unexplained END” (3).

Perfusion imaging has been confirmed to be an effective aid in the decision-making of reperfusion therapy (4). A previous study showed that penumbra/infarct growth beyond the initial penumbra might lead to unexplained END in patients with AIS (5). Moreover, patients who are presenting hypoperfusion in pure lenticulostriate arteries (LSAs) territory were more likely to experience unexplained END (6). In addition, weighted corticospinal tract lesion load, a biomarker reflecting the degree and volume of corticospinal tract (CST) infarction of AIS, was reported to be well correlated with poststroke motor outcomes at 3 months (7). Tissue microstructure alterations in CST were closely associated with the thickness of the primary motor cortex in patients with brain injury (4, 8). However, it remains unknown whether baseline hypoperfusion in CST territory was related to unexplained END in patients treated with IVT. Therefore, we aimed to explore whether the presence of baseline CST hypoperfusion could predict the development of unexplained END at 24 h in patients with AIS after receiving IVT.

## Materials and Methods

### Study Design

We retrospectively analyzed a prospectively collected database of patients with AIS who received IVT from May 2009 to May 2020. Patients were enrolled if they satisfied the following criteria: (1) received IVT within 9 h after the onset; (2) underwent baseline CT perfusion (CTP) before IVT. Patients were excluded if they had the following conditions: (1) had cerebral parenchymal hemorrhage (PH) within 24 h, which was defined as a hematoma in ≤ 30% of the infarcted area with some slight space-occupying effect or blood clots in > 30% of the infarcted area with a substantial space-occupying effect (9). (2) Had malignant brain edema within 24 h, which was defined as concomitant imaging which showed brain swelling and midline shift which were associated with worsening of consciousness (5). (3) Had early recurrent stroke within 24 h, which was defined as the new neurological symptoms along with the evidence of corresponding ischemic lesions on follow-up cranial CT or MRI, which suggested the involvement of initially unaffected vascular area (5). (4) Finally, if they had failed image post-processing due to movement artifacts or huge previous lesions.

### Ethics Statement

All subjects had given written informed consent prior to the study, and the protocols had been approved by the local human ethics committee. The clinical investigation had been conducted according to the principles expressed in the Declaration of Helsinki.

### Imaging Protocol

Computed tomography perfusion was performed on a dual-source 64 slices CT scanner (SOMATOM Definition Flash; Siemens, Forchheim, Germany), including non-enhanced CT head scan (120 kV, 320 mA, contiguous 5 mm axial slices) and volume perfusion CT (100 mm in the z-axis, 4 s delay after start of contrast medium injection, 74.5 s total imaging duration, 80 kV, 120 mA, effective dose = 3.68 mSv, slice thickness 10 mm, and collimation 32 × 1.2 mm). Furthermore, a 60 ml contrast medium (Iopamidol; Braccosine, Shanghai, China) was used at a flow rate of 6 ml/s, followed by a 20 ml saline tracker at a flow rate of 6 ml/s. Axial, coronal, and sagittal planes image were reconstructed by CTP, with a maximum density projection thickness of 20 mm. CTP examination was routinely available in patients with AIS within 24 h of the disease's onset if there is no contraindication after 2011.

### Imaging and Clinical Analysis

**Figure 1:**
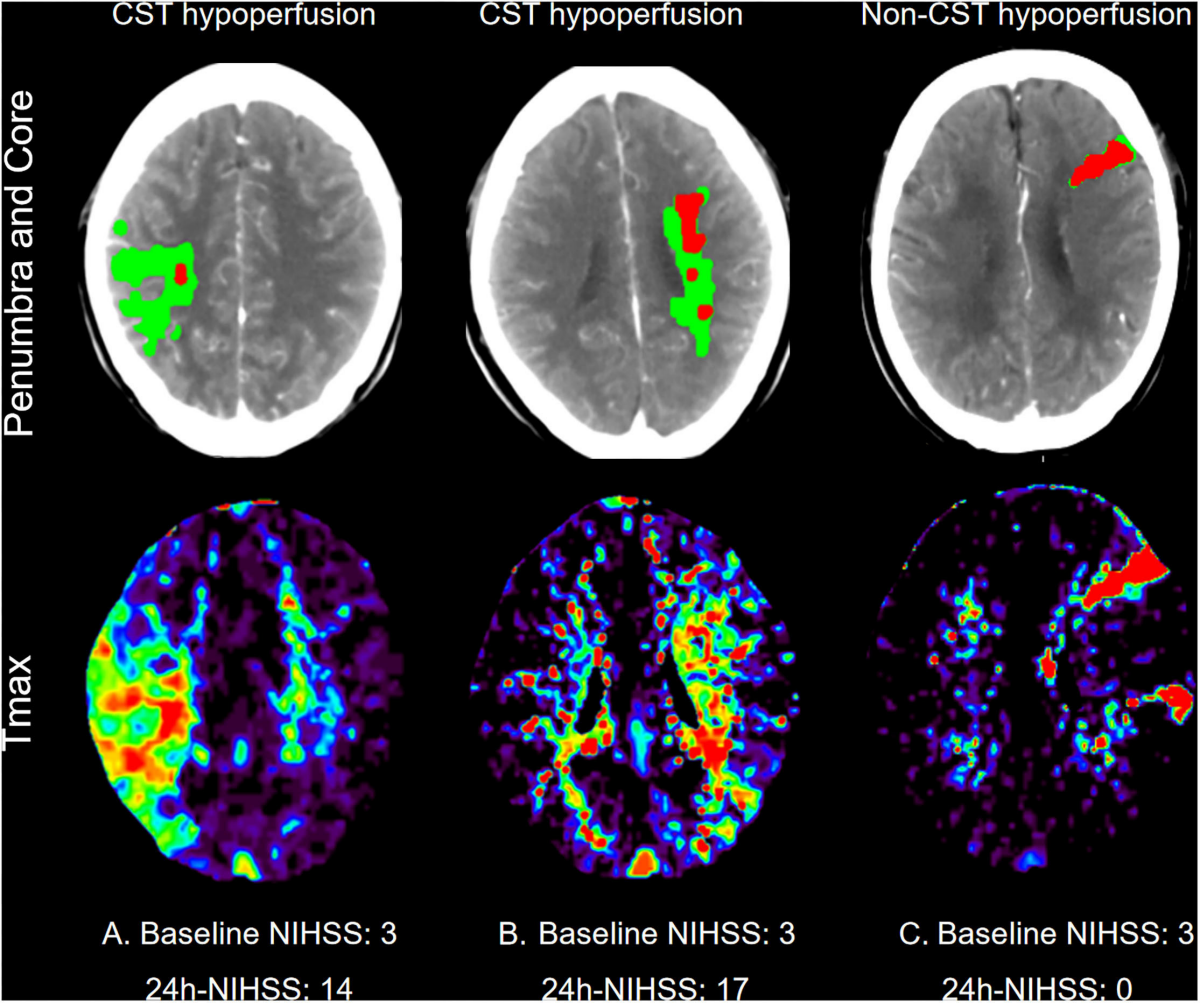
Representative examples of patients who underwent computed tomography perfusion at baseline. First line: Penumbra (Green Area) and core (Red Areas) Map; Second line: Time to maximum of tissue residue function (Tmax) map. **(A)** One patient with CST hypoperfusion at right corona radiata, baseline NIHSS: 3; 24h-NIHSS: 14. **(B)** One patient with CST hypoperfusion at left lateral ventricle, baseline NIHSS: 3; 24h-NIHSS:17. **(C)** One patient with non-CST hypoperfusion, baseline NIHSS: 3; 24h-NIHSS: 0.

All perfusion images were retrospectively post-processed on the Siemens workstation (Syngovia VB20A, Siemens Healthcare GmbH, Erlangen, Germany). The time to a maximum of tissue residue function (Tmax) map was produced using standard singular value deconvolution without delay-correction. Hypoperfusion was defined as Tmax > 6 s on CTP at baseline. Two experienced neurologists (DZ and CX) examined all perfusion images and identified the site of CST in the corresponding layers on post-processed CT images, and whether hypoperfusion involved CST areas, including corona radiata, central semi-elliptic, lateral ventricle, the posterior limb of the internal capsule, and brainstem area. CST hypoperfusion was defined as hypoperfusion involving CST areas. Non-CST hypoperfusion was defined as hypoperfusion involving areas other than CST and no hypoperfusion (Figure 1). Unexplained END was defined as ≥ 2-point increase of National Institutes of Health Stroke Scale (NIHSS) from baseline to 24 h, without straightforward causes.

### Statistical Analysis

Clinical characteristics were summarized as mean ± *SD* or median (25th−75th percentile) for quantitative variables and as proportions for categorical variables. Chi-square test was used to compare the dichotomous variables between groups, whereas independent samples two-tailed *t*-test or Mann–Whitney *U*-test was used for the continuous variables, as appropriate. Binary logistic regression analysis was used to generate odds ratios. Variables with a *P* < 0.1 in univariate analyses were included in the multivariate analysis. A *P* < 0.05 was considered to be statistically significant.

## Result

**Figure 2:**
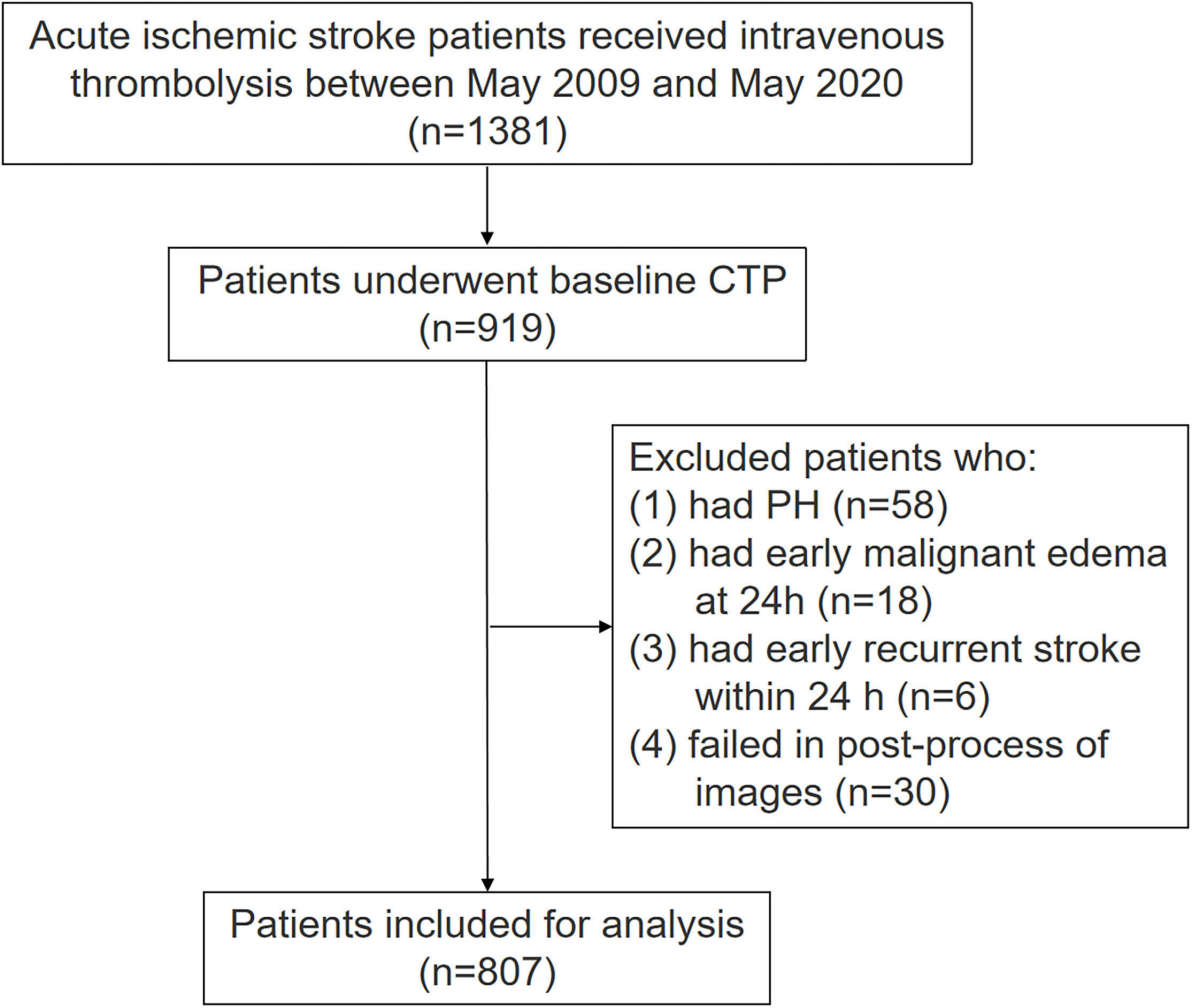
Patients flow chart.

Among 1,381 patients with AIS who received IVT, 919 of them underwent baseline CTP. In our center, multimodal MRI but not CTP was available before 2011. After 2011, CTP was routinely used in patients with AIS within 24 h of the disease's onset if there is no contraindication, but some stroke doctors still preferred to use multimodal MRI. The current analysis included all patients from 2009 since our prospectively collected database of patients with AIS receiving IVT was initiated from May 2009. Among 462 patients with AIS without CTP examination, 189 of them performed non-contrast computerized tomography (NCCT), 230 patients performed MRI, and 43 patients already performed image examination in other hospitals before admission. Among 919 patients, we further excluded 112 patients due to the following reasons: (1) they had PH within 24 h (*n* = 58); (2) they had malignant brain edema within 24 h (*n* = 18); (3) they had early recurrent stroke within 24 h (*n* = 6); (4) they failed to image post-processing (*n* = 30). The final analysis includes 807 patients. The flow chart of patient selection is illustrated (Figure 2). Among the included patients, mean age was 69 ± 13 years and 298 (36.9%) were women, median NIHSS was 6 (3–12), median onset to needle time (ONT) was 203 min (140–270 min), and 488 (60.5%) and 319 (39.5%) patients presented CST hypoperfusion and non-CST hypoperfusion, respectively. Finally, 166 out of 319 (52%) patients had no hypoperfusion. Unexplained END was present in 90 patients (11.2%).

As Table 1 shows, compared with patients with non-unexplained END, those with unexplained END had a higher rate of hypertension and higher baseline hypoperfusion volume (all *P* < 0.05). There was no significant difference in gender, age, smoking, previous stroke/TIA, diabetes mellitus, or atrial fibrillation between patients with and without unexplained END (all *P* > 0.05).

**Table 1:** Comparison between unexplained early neurological deterioration (END) and non-unexplained END.

**Characteristic**	**Unexplained END (*n* = 90)**	**Non-unexplained END (*n* = 717)**	***P*-value**
Age (year), mean ± SD	67 ± 13	69 ± 13	0.164
Female, n (%)	32 (35.6)	266 (37.1)	0.817
Baseline hypoperfusion Volume (mL), median (IQR)	51.9 (1.7–101.5)	13.2 (0–47.0)	0.001
Baseline NIHSS, median (IQR)	6 (3–11)	6 (3–12)	0.010
ONT, min, median (IQR)	227 (164–282)	197 (135–270)	0.001
**Risk factors**			
Smoking, *n* (%)	39 (43.3)	260 (36.3)	0.204
Hypertension, *n* (%)	70 (77.8)	481 (67.1)	0.041
Previous stroke or TIA, n (%)	20 (22.2)	131 (18.3)	0.389
Diabetes mellitus, *n* (%)	24 (26.7)	165 (23.0)	0.431
Atrial fibrillation, *n* (%)	27 (30.3)	220 (30.7)	1.000
No hypoperfusion, *n* (%)	6 (6.7)	160 (22.3)	<0.001
CST Hypoperfusion, *n* (%)	81 (90.0)	407 (56.8)	<0.001

*IQR, interquartile range; NIHSS, National Institutes of Health Stroke Scale; ONT, onset to treatment time; TIA, transient ischemic attack; CST, corticospinal tract; END, early neurological deterioration*.

Table 2 shows that patients with CST hypoperfusion had higher baseline NIHSS, baseline hypoperfusion volumes, longer ONT, higher rate of hypertension, atrial fibrillation, and unexplained END compared with patients with non-CST hypoperfusion (all *P* < 0.05). Patients with CST hypoperfusion were more likely to experience unexplained END than patients with non-CST hypoperfusion (16.6 vs. 2.8%, *P* < 0.001).

**Table 2:** Comparison between patients with corticospinal tract (CST) and non-CST hypoperfusion.

**Characteristic**	**CST hypoperfusion (*n* = 488)**	**Non-CST hypoperfusion (*n* = 319)**	***p*-value**
Age (year), mean ±SD	70 ± 14	68 ± 12	0.113
Female, *n* (%)	184 (37.7)	114 (35.7)	0.602
Baseline hypoperfusion volume (mL), median (IQR)	38.5 (10.0–84.0)	0 (0–11.0)	<0.001
Baseline NIHSS, median (IQR)	8 (4–14)	5 (3–9)	<0.001
ONT (min), median (IQR)	216 (150–287)	185 (125–248)	<0.001
**Risk factors**			
Smoking, *n* (%)	179 (36.7)	120 (37.6)	0.823
Hypertension, *n* (%)	351 (71.9)	200 (62.7)	0.007
Previous stroke or TIA, *n* (%)	102 (20.9)	49 (15.4)	0.053
Diabetes mellitus, *n* (%)	121 (24.8)	68 (21.3)	0.270
Atrial fibrillation, *n* (%)	171 (35.0)	76 (23.8)	0.001
Unexplained END, *n* (%)	81 (16.6)	9 (2.8)	<0.001

*CST, corticospinal tract; IQR, interquartile range; NIHSS, National Institutes of Health Stroke Scale; ONT, onset to needle time; TIA, transient ischemic attack; END, early neurological deterioration*.

After adjusting for baseline NIHSS, hypertension, atrial fibrillation, previous stroke or TIA, ONT, and baseline hypoperfusion volume, the presence of CST hypoperfusion was independently associated with unexplained END [odds ratio (OR) = 5.64; 95% CI: 2.699–11.785; *P* < 0.001]. In addition, baseline NIHSS (OR = 0.913; 95% CI:0.872–0.955; *P* < 0.001) and baseline hypoperfusion volume (OR = 1.009; 95% CI: 1.006–1.013; *P* < 0.001) were also independently associated with unexplained END (Table 3). We also analyzed the results after excluding patients with no hypoperfusion. This analysis included 641 patients. We found that CST hypoperfusion was still independently associated with unexplained END after IVT (96.4 vs. 73.1%, OR = 9.021; 95% CI: 2.738–29.721; *P* < 0.001) after adjusting for baseline NIHSS, onset to needle time, baseline hypoperfusion volume, atrial fibrillation, and hypertension.

**Table 3:** Multivariable analysis for unexplained early neurological deterioration.

**Variable**	**Odds ratio**	**95% CI**	***P-*value**
Baseline NIHSS	0.913	0.872–0.955	<0.001
ONT	1.001	0.999–1.002	0.424
Baseline hypoperfusion volume	1.009	1.006–1.013	<0.001
Atrial fibrillation	0.874	0.517–1.479	0.617
Hypertension	1.543	0.887–2.684	0.125
CST Hypoperfusion	5.675	2.717–11.858	<0.001

*NIHSS, National Institutes of Health Stroke Scale; ONT, onset to needle time; CST, corticospinal tract*.

## Discussion

In this study, 11.2% of patients with AIS had unexplained END after IVT. Moreover, we found that patients with AIS along with CST hypoperfusion had a higher risk to experience unexplained END than patients with non-CST hypoperfusion.

Our findings of the relationship between CST hypoperfusion and unexplained END in patients with AIS receiving IVT were consistent with previous findings. CST is a major neural tract for motor function in the human brain, supplied by perforating arteries with little collateral flow. The study Zhou et al. (6) suggested that hypoperfusion in LSAs territory was related to unexplained END after IVT. Branch atheromatous disease (BAD) and small arteries occlusion (SAO) were two possible pathologies of pure LSA territory stroke, which were prone to suffer END. Moreover, the maximum ventrodorsal length multiplied by rostrocaudal thickness in lesion planes of acute pontine infarction patients, which was related to ischemic damage of the corticospinal tract and corticobulbar tract, was significantly associated with END (10).

In addition, we found that patients with higher baseline NIHSS and hypoperfusion volume were more likely to experience unexplained END. The previous study also showed that initial hypoperfusion and admission NIHSS were strongly associated with unexplained END, which is consistent with our finding (2).

Branch atheromatous disease was strongly associated with motor deficits induced by a stroke in the lenticulostriate artery and anterior pontine artery areas, consequently leading to worse functional outcomes (11). Thromboembolus was disintegrated and fall off after IVT, forming local thrombus or prolonging *in situ* thrombus, which was easy to occlude perforating vessel supplying CST. Due to the lack of collateral flow, the hypoperfusion of CST was difficult to be reperfused once perforating artery was occluded. Therefore, CST hypoperfusion, along with local blood-brain barrier failure, local thrombosis, inflammation, edema, and excitotoxicity, may be the potential causes for AIS progression (12). In addition, prolongation of thrombus and collateral status were associated with END, which further implied that hypoperfusion involving the CST might lead to unexplained END (13, 14).

Currently, a significant proportion of patients with AIS are present with unexplained END after IVT (about 11.2%). Thus, timely and accurate prediction for potential unexplained END is necessary to prevent poor outcomes in thrombolytic patients as early as possible. Antiplatelet therapy can improve the functional recovery of patients with AIS (15, 16). Moreover, prior antiplatelet use may reduce initial stroke severity and the risk of END (17). It might be possible to select patients with AIS along with CST hypoperfusion to apply intensified antiplatelet therapy after IVT in order to reduce unexplained END. Future clinical trials are therefore required to confirm it. In addition, the ARAIS trial has been conducted to evaluate whether argatroban plus rt-PA is superior to rt-PA alone in improving functional outcomes in patients with AIS (18). A recent study indicated that argatroban reduced the risk of END in AIS after reperfusion therapy (19). Thus, CST hypoperfusion might be a potential indication to explore whether argatroban plus rt-PA could reduce unexplained END.

There are some limitations to this study. Firstly, although we prospectively collected data, our study had a retrospective design and had a potential risk of selection bias. Secondly, small lesions might not be assessed on perfusion images, which might also influence the clinical outcome. Thirdly, given that new occlusion of relatively small arteries was challenging to be identified on MRA or CTA in our study, we could not make sure that all the cases with early recurrent stroke had been excluded.

## Conclusion

This study concluded that patients with AIS along with CST hypoperfusion are more likely to experience unexplained END after IVT, which may provide a clue for future investigation of potential novel approaches to prevent post-thrombolysis unexplained END.

## Data Availability Statement

The raw data supporting the conclusions of this article will be made available by the authors on reasonable request, without undue reservation.

## Ethics Statement

The studies involving human participants were reviewed and approved by the Ethics Committee of the Second Affiliated Hospital of Zhejiang University, School of Medicine. The patients/participants provided their written informed consent to participate in this study.

## Author Contributions

ML, DZ, WZ, SY, and YZ: conceptualization. DZ and WZ: formal analysis and software. ML, DZ, WZ, and CX: methodology. ML, DZ, LC, CX, and XM: supervision. LC, YZ, and SY: validation. DZ, WZ, and LC: writing-original draft preparation. ML and WZ: writing-review and editing. All authors have read and agreed to the published version of the manuscript. All authors contributed to the article and approved the submitted version.

## Funding

This study was supported by the National Natural Science Foundation of China (81971101, 82171276) and the Science Technology Department of Zhejiang Province (2018C04011).

## Conflict of Interest

The authors declare that the research was conducted in the absence of any commercial or financial relationships that could be construed as a potential conflict of interest.

## Publisher's Note

All claims expressed in this article are solely those of the authors and do not necessarily represent those of their affiliated organizations, or those of the publisher, the editors and the reviewers. Any product that may be evaluated in this article, or claim that may be made by its manufacturer, is not guaranteed or endorsed by the publisher.
